# An evolving perspective on physical activity counselling by medical professionals

**DOI:** 10.1186/1471-2296-13-31

**Published:** 2012-04-23

**Authors:** Steven McPhail, Mandy Schippers

**Affiliations:** 1 School of Public Health and Institute of Health and Biomedical Innovation, Queensland University of Technology, Brisbane, Queensland, Australia; 2 Centre for Functioning and Health Research, Queensland Health, Brisbane, Queensland, Australia

## Abstract

**Background:**

Physical inactivity is a modifiable risk factor for many chronic conditions and a leading cause of premature mortality. An increasing proportion of adults worldwide are not engaging in a level of physical activity sufficient to prevent or alleviate these adverse effects. Medical professionals have been identified as potentially powerful sources of influence for those who do not meet minimum physical activity guidelines. Health professionals are respected and expected sources of advice and they reach a large and relevant proportion of the population. Despite this potential, health professionals are not routinely practicing physical activity promotion.

**Discussion:**

Medical professionals experience several known barriers to physical activity promotion including lack of time and lack of perceived efficacy in changing physical activity behaviour in patients. Furthermore, evidence for effective physical activity promotion by medical professionals is inconclusive. To address these problems, new approaches to physical activity promotion are being proposed. These include collaborating with community based physical activity behaviour change interventions, preparing patients for effective brief counselling during a consultation with the medical professional, and use of interactive behaviour change technology.

**Summary:**

It is important that we recognise the latent risk of physical inactivity among patients presenting in clinical settings. Preparation for improving patient physical activity behaviours should commence before the consultation and may include physical activity screening. Medical professionals should also identify suitable community interventions to which they can refer physically inactive patients. Outsourcing the majority of a comprehensive physical activity intervention to community based interventions will reduce the required clinical consultation time for addressing the issue with each patient. Priorities for future research include investigating ways to promote successful referrals and subsequent engagement in comprehensive community support programs to increase physical activity levels of inactive patients. Additionally, future clinical trials of physical activity interventions should be evaluated in the context of a broader framework of outcomes to inform a systematic consideration of broad strengths and weaknesses regarding not only efficacy but cost-effectiveness and likelihood of successful translation of interventions to clinical contexts.

## Background

Physical inactivity is a modifiable risk factor for a multitude of chronic conditions including heart disease, stroke, diabetes mellitus, depression and a range of musculoskeletal disorders [1-7]. Individuals who engage in regular moderate physical activity are less likely to die prematurely [8-11]. In the U.S., physical inactivity now closely follows tobacco use as the leading effective cause of death [12-14]. Approximately 250,000 deaths could be prevented in the United States each year if citizens were to become moderately physically active [15]. In addition to personal health impacts, physical inactivity also places a considerable financial burden on individuals and the economy as a whole. In 2006, the cost of physical inactivity to the U.S. economy was estimated at $251 billion [16]. Negative impacts of physical inactivity are not limited to the U.S. population. The World Health Organisation (WHO) has identified physical inactivity as the fourth leading effective cause of death globally [17].

There are a range of benefits associated with increasing physical activity [1,4-7,18-20]. Immediate gains include improved musculoskeletal and mental health as well as cardiovascular and respiratory benefits [18]. Medium and longer term benefits are wide ranging. Physical activity reduces the severity of existing health conditions and can prevent a range of further co-morbidities [4,6,7,19]. In patients with multiple chronic conditions, physical activity has been associated with a reduced 42-month all-cause mortality rate [20]. Among people who are obese, becoming physically active can reduce the chance of chronic health conditions to the level of people who are within a healthy-weight range but are not physically active [5]. Benefits are not limited to youth or young adults. Resistance exercise training among octogenarians can improve muscle strength, mobility, dynamic balance, reduce falls and lead to more spontaneous physical activity [21,22].

The benefits of physical activity are dependent on the intensity, duration and frequency with which it is undertaken. The American College of Sports Medicine and the American Heart Foundation recommend a minimum of five 30-minute sessions of moderate intensity aerobic activity (e.g. brisk walking or equivalent) or three 20-minute sessions of vigorous intensity aerobic activity (or a combination of both) [23]. However, it is noteworthy that reducing sedentary time, regardless of how much time is spent in moderate or vigorous physical activity, has also been associated with reduced mortality [24].

Despite the known benefits of physical activity, a large proportion of people in developed and developing nations do not meet minimum recommendations for physical activity [25]. More than 95% of adults in the United States do not meet the recommended level of 30 minutes of moderate intensity physical activity per day [26]. More than 25% are completely sedentary during their leisure time [25]. Many explanatory factors have been proposed; most relate to societal changes during recent decades that have contributed to the commonality of sedentary lifestyles [27-32]. These factors include the increased popularity of sedentary behaviours in recreational and social pursuits, educational settings and occupational activities [30,31]. This transition to sedentary lifestyles has been accelerated by the rapid advancement of information technologies used in recreation, education and occupational contexts [27-29,32].

The burden of physical inactivity will increase as the population ages. Between 2000 and 2040 the U.S. Census Bureau predicts an increase in the number of citizens older than 84 years from 3.5 million to 8–13 million [33]. This represents a doubling or tripling of this age group. This substantial growth in older adults will not be offset by equivalent growth in younger age groups. The increasing relative proportion of elderly people is not limited to the U.S. but is occurring throughout developed nations [33]. The increase will continue to accelerate after 2011 when the first of the baby boom generation will reach 65 years of age [33].

Many diseases that are considered to be related to aging (e.g. cardiovascular disease, stroke, musculoskeletal disorders) can be prevented or alleviated through regular physical exercise [34]. This intensifies the need for suitable physical activity behaviour change interventions targeted at priority groups. Targeting effective physical activity behaviour change interventions to priority population groups will not only minimise the personal impacts of inactivity. It will also reduce the extensive financial burden of healthcare costs associated with the sequelae of physical inactivity.

Medical professionals have been identified as having the potential to be powerful agents for physical activity promotion [35-43]. During the course of their existing work they reach a large proportion of the population [44-47]. Research from the United Kingdom indicates 90% of the population visit their primary care physician at least once within each three year period [48]. Moreover, visits to a medical professional become more frequent as people age [37]. Medical professionals are also respected sources of preventive health promotion with patients listing their primary care physician as a desired and expected provider of preventive care information [37,39,41]. Furthermore, messages from medical professionals can have a catalysing effect on motivating change in exercise-related health behaviours [36,40,42,43]. This effect is not isolated to physical activity and has been observed in other health behaviours; such as smoking cessation [49,50].

The potential for medical professionals to impact physical activity behaviours amongst their patients has led to repeated recommendations for physical activity promotion to be incorporated into routine clinical practice [35,38,51]. The U.S. Preventive Services Task Force (USPSTF) is an independent panel of prevention and evidence-based medicine experts, composed of primary care providers [52]. The USPSTF conducts scientific evidence reviews and published its first report on clinical preventive services in 1989 [52,53]. The report stated that clinicians will be more effective if they address the health behaviours of their patients rather than by performing usual screening tests and physical examinations [53]. To illustrate this point, epidemiological calculations have indicated that 205 45-year old women would be required to undergo mammography screening to prevent one premature death [54]. This is in contrast to one premature death being prevented for each 16 of these women who become sufficiently physically active [54]. Recommendations for primary care providers to incorporate physical activity counselling into their routine practice have been echoed by other organisations in the policy community (e.g. the American College of Preventive Medicine in 2005 [51] and the Australian Heart Foundation in 2006) [35].

Despite these recommendations, medical professionals still incorporate very little physical activity promotion into their routine practice [55-59]. In one study among a diverse sample of U.S. adults, only 28% of respondents reported receiving advice about physical activity from a physician [55]. Of these respondents, less than half received help with formulating an activity plan or follow-up support [55]. It is clear that existing approaches to the management of inactive patients in day to day practice are flawed. In this paper we examine whether physical activity promotion by medical professionals is feasible and effective as part of their routine care for inactive patients and we discuss an alternative perspective on this role. To inform this discussion we have considered peer reviewed empirical research as well as recommendations or statements from government and health organisations.

## Discussion

### Medical professionals face barriers to physical activity promotion

Medical professionals value their role as promoters of healthy behaviours in their patients [60-62]. However, they have consistently experienced a number of barriers to providing health behaviour counselling [62-66]. They perceive their patients to be uninterested in increasing their physical activity levels and unlikely to change their behaviour [57,63,67]. They also lack confidence in their counselling skills [57]. When medical professionals do devote time to counselling, they do not usually receive positive feedback from patients becoming more physically active [68-70].

Perhaps the greatest barrier to physical activity counselling by medical professionals is the limited consultation time available for each patient [63]. It has been estimated that complying with all recommendations laid out by the USPSTF would require medical professionals to spend approximately 7.5 hours of every working day on prevention activities [66]. Research from the U.S. indicates that physicians currently devote an average of 42 seconds per patient encounter when undertaking health behaviour counselling with patients [71]. Even ‘brief counselling’ in successful physical activity promotion interventions requires at least 3 to 5 minutes [64,71]. While 3 to 5 minutes per visit does not sound like a large time demand, it has been proposed that this may be unrealistic in some busy clinical environments [65]. For physicians this would comprise 30% to 50% of a typical patient visit [64,71]. Under existing funding models, medical professionals are not reimbursed for incorporating physical activity counselling into their busy schedules [25].

### Could physical activity counselling by medical professionals be effective?

Systematic literature reviews of the effectiveness of physical activity counselling by medical professionals have concluded that evidence is mixed at best [64,72,73]. This is primarily due to heterogeneous interventions and methodological shortcomings across clinical trials [64,72,73]. Several studies have shown positive effects of physical activity counselling by medical professionals [74,75]. However, effect sizes have been small and there is little evidence for long-term effects on physical activity [64,72,73]. Furthermore, little is known about what elements of physical activity promotion are associated with its effectiveness (e.g. the length and number of counselling sessions, the theoretical basis of the intervention) [72]. Based on these findings the USPSTF have recently changed their recommendation and currently state that evidence is insufficient to warrant any recommendations for or against physical activity counselling by medical professionals [73,76].

It is also conceivable that the broad uptake of physical activity counselling by medical professionals could be associated with some drawbacks. It is possible that patients may perceive interaction with their treating medical professional as a negative experience if they are frequently reminded of their inactive lifestyle. For example, a physically inactive diabetes patient may be reluctant to visit his doctor to discuss his problematic blood glucose levels knowing he will likely be required to again report he has not followed prior advice to become more active. This may lead to an avoidance of primary care physicians and a delay in treatment for emerging complications or other health conditions. It is also plausible that patients of medical professionals who undertake physical activity counselling will incur increased out of pocket costs associated with longer consultation times.

Despite these potential drawbacks, several authors suggest that medical professionals should play a lead role in physical activity promotion provided that key barriers (such as insufficient time) are overcome [47,65,77,78]. There are several alternative opportunities to augment physical activity counselling by medical professionals. These include use of community based interventions, preparing patients for referral to external physical activity interventions and use of interactive technologies [78-89].

### Community based interventions

Medical professionals may not need to perform extensive counselling to be able to assist patients with increasing their physical activity. They are identified as potentially strong promoters of physical activity because they are respected, trustworthy and expected sources of advice and are regularly visited by a large proportion of the population [37,39,41,44-47]. Thus, medical professionals could use their limited time and resources for providing patients with brief, personally relevant and timely health promotion messages and referring them to external sources for more comprehensive community-based support.

Community based interventions usually include a combination of risk factor screening, health education, counselling and social support [90]. They may be based in a variety of community settings including health centres, not-for-profit health organisations, hospital ambulatory settings, workplaces, universities or schools [79-83,90-93]. They may also involve communication techniques in various media formats aimed at reaching many people with minimal cost [90]. Community based interventions may be suitable for a wide range of people or targeted to a specific group. One example includes the MobileMums program [94]. This intervention is targeted to postnatal (<12 months postpartum) women. The MobileMums program includes a 12 week intervention commencing with a face-to-face goal setting interview. Support is offered via personally tailored mobile telephone short message service (SMS) sent to the individual and to a nominated support person. This is just one of many community based programs that have been identified as cost-effective and successful in improving physical activity levels [90-92].

Moving the bulk of the behaviour change intervention to community services outside of an existing consultation may improve its effectiveness [79-83]. The effectiveness of physical activity promotion by medical professionals is enhanced when systems are in place that support all necessary steps in a counselling process [47]. These steps include assessment of patients’ health behaviour status and readiness to change, advice on behaviour change, agreement on a course of action, assistance with the changes in health behaviour, and arranging further help where needed [47]. These elements are part of the 5-A’s framework that has been effectively used in the development of health promotion interventions [47].

Community based interventions can be easily integrated into patients’ ongoing management through feedback to the referring medical professional. Interventions can incorporate feedback to medical professionals regarding physical activity behaviour changes. This feedback enables medical professionals to see the effects of their health promotion efforts and potentially act as a mode of positive reinforcement when favourable changes have been achieved. Additionally, this feedback may influence related health intervention decisions (such as diabetes management plans). Importantly, if medical professionals need only to refer patients to external support rather than provide counselling, this may reduce the consultation time required to successfully increase the physical activity levels of their patients. Reduced consultation time demand and the potential for positive feedback may contribute to improved health promotion self-efficacy among medical professionals. This would likely result in physical activity promotion to a higher proportion of inactive patients.

### Preparing patients for referral to physical activity interventions

Community based interventions allow medical professionals to move most elements of a comprehensive behaviour change intervention outside of their consultation time [77]. This may reduce the barriers they experience with counselling patients. However, this approach is dependent on the medical professional’s ability to engage patients in undertaking community based physical activity interventions. To this end, medical professionals may foster awareness when patients are not sufficiently active and prepare them for undertaking behaviour change to address this deficit. There are several time efficient strategies medical professionals may use to optimise their success in referring inactive patients to physical activity behaviour change interventions. These strategies include preparing patients before the consultation, completing health risk assessments and using teachable moments or brief motivational interviewing during their consultations [84-89,95].

Preparing patients before a consultation with their medical professional may increase the effectiveness of efforts to address the topic of physical inactivity. Receiving information or advice related to health behaviours improves patients’ response to subsequent educational information [88,89]. Patients could receive information about the topic in the waiting room using information sheets or posters, or before the visit via a phone call or letter. Additionally, short patient assessments or screening before the visit may further facilitate a concise discussion of physical activity between inactive patients and medical professionals [87]. The results of the assessment may help the medical professional to address the topic of physical activity during the patient encounter. Health risk assessment can be used to draw attention to the potential health risks associated with a patient’s lifestyle to reinforce the health promotion message [87]. Short physical activity stages of change questionnaires that can be self-completed by patients in the waiting room are also available [95]. The readiness to change information used in conjunction with a health risk assessment may assist medical professionals to tailor the message delivered to the patient during the visit and inform the type of external support the patients is referred to. This may be further facilitated by the use of behaviour change techniques during the consultation.

Two practical techniques that can be applied during the consultation include teachable moments and brief motivational interviewing. Teachable moments may enable medical professionals to deliver health promotion information in a constructive way [86]. These are moments during an interaction between a medical professional and a patient where there is an opportunity to promote health behaviour change [65,96-101]. These opportunities often arise out of circumstances in the patient’s life that can be related to health behaviour change [99,102]. Using teachable moments can lead to more cooperation and patient initiative to change negative health behaviour [86]. Teachable moments occur naturally in approximately 10% of physician-patient conversations [86]. A pre-visit screening for health risks will likely provide additional opportunities to address physical activity behaviours among inactive patients.

Another potentially useful technique to assist medical professionals is motivational interviewing. Motivational interviewing has been successfully applied in several areas of health-related behaviour change to bring people closer to initiating positive behaviours [84,85]. Conventional motivational interviewing can be time intensive [103]. However, brief motivational interviewing has been developed for clinicians who can devote little time to receiving training and delivering counselling [104]. Brief motivational interviewing offers a range of strategies suitable for people in the different stages of readiness to change [86,104].

### Interactive behaviour change technology

Whether behaviour change interventions are delivered in conjunction with medical professional consultations or entirely from community based programs, efficient use of time and resources will increase the feasibility of their implementation. Contemporary information technology can reduce the time and cost involved in providing physical activity interventions. A variety of platforms have been used to successfully automate components of physical activity behaviour change interventions [78]. These platforms include Personal Digital Assistants (PDAs), mobile telephones and other handheld devices, interactive web sites, computer surveys, and interactive voice response technology (automated telephone calls) [78,105]. This array of platforms has been referred to as Interactive Behaviour Change Technology (IBCT) [78].

IBCT can be used to streamline several operational processes during health promotion activities. Automatic assessment of participants before referral to health promotion interventions can reduce time otherwise spent on administering assessments, scoring assessments, selecting intervention materials and monitoring progress [106,107]. During external behaviour change interventions, participant progress can be monitored through regular technology based assessment of relevant behaviours, health-related outcomes or problems experienced with the intervention [108,109]. Continuing rapid progression in IBCT will likely result in further efficiency gains in the implementation of physical activity behaviour change interventions among inactive patients.

## Summary

### The physical inactivity problem

Physical inactivity is a modifiable risk factor for various chronic conditions and an important contributing factor to premature mortality [2-7]. Medical professionals have been identified as having the potential to positively influence the physical activity levels of a large proportion of the population [35-43]. Medical professionals value their role as promoters of physical activity among their inactive patients. However, they experience several barriers to incorporating physical activity promotion into routine clinical practice [62-66]. Despite repeated recommendations, medical professionals infrequently deliver messages of physical activity promotion to their inactive patients [56-59].

As the population ages, the negative impact of physical inactivity is likely to compound [33,34]. The need to increase physical activity at a population level will become increasingly urgent. If medical professionals are to reach their potential to positively influence physical activity behaviours amongst their inactive patients, a new perspective on their role is necessary. This new perspective must take into account the barriers they experience in day-to-day clinical practice [47,65,77,78].

### A new perspective for medical professionals

It is important that we recognise the latent risk of physical inactivity among patients presenting in our clinical settings. We must also consider the positive long-term effects of addressing physical inactivity and consider this a key component of treatment for inactive patients. Preparation for improving patient physical activity behaviours should commence before the consultation. Health risk assessments or physical activity screening questionnaires can be self-completed by most patients in the waiting room or prior to attendance. To this end, medical professionals may choose to change the management of their clinical practice to incorporate screening of patients and provision of written information before the consultation. Medical professionals must also identify suitable community interventions to which they can refer physically inactive patients. Outsourcing the majority of a comprehensive physical activity intervention to community based interventions will reduce the required clinical consultation time for addressing the issue with each patient.

### Priorities for research

There are several key priorities for research in this field. Factors that promote engagement in community based physical activity behaviour change interventions are worthy of investigation. This should include research to optimise the success rate of referral to a physical activity intervention delivered outside of routine clinical consultations. For example, research is needed to determine which screening techniques are most successful in enhancing a subsequent referral by a medical professional to external physical activity counselling. Future clinical trials investigating the effectiveness of physical activity behaviour change interventions should be delivered in real world contexts to ensure findings represent the likelihood of success in day-to-day practice. These clinical trials may benefit from factorial designs to help identify intervention parameters that optimise intervention effectiveness and logical combinations of intervention elements. Parameters to be investigated should include intervention timing, intensity, duration and mode of delivery. This includes investigating which elements of IBCT are most effective and how these technologies can be incorporated to streamline intervention implementation. Research in this field should not only include inactive people who are otherwise healthy, but the translation of physical activity interventions to priority patient groups. This includes patients with existing health conditions who are likely to experience complications or other chronic conditions associated with inactivity. Finally, future research should assess physical activity interventions in the context of a broader framework of outcomes to inform a systematic consideration of broad strengths and weaknesses regarding not only efficacy but cost-effectiveness and likelihood of successful translation to clinical practice.

## Competing interests

The authors declare that they have no competing interests.

## Authors' contributions

SM and MS contributed to the design, planning, drafting, appraisal and editing of the manuscript. Both authors read and approved the final manuscript.

## Pre-publication history

The pre-publication history for this paper can be accessed here:

http://www.biomedcentral.com/1471-2296/13/31/prepub
